# Transcriptomics Implicates the G3BP1–SG Axis in Cochlear Hair‐Cell Pyroptosis During Age‐Related Hearing Loss

**DOI:** 10.1111/jcmm.71403

**Published:** 2026-10-11

**Authors:** Chen Li, Yuan Cao, Jun Zhou, Yurun Chen, Qi Tang, Shuyi Wang, Zhiyuan Wu, Shu Wang, Zixin Zhang, Mengyao Xie, Hong Huo, Hua Yang

**Affiliations:** ^1^ Department of Otolaryngology Peking Union Medical College Hospital, Chinese Academy of Medical Sciences & Peking Union Medical College Beijing China; ^2^ Eight‐Year Medical Doctor Program Chinese Academy of Medical Sciences & Peking Union Medical College Beijing China; ^3^ Clinical Biobank (ISO 20387) Peking Union Medical College Hospital, Chinese Academy of Medical Sciences Beijing China

## Abstract

Age‐related hearing loss (ARHL) is a degenerative change in the auditory system, but its underlying mechanism remains unclear. In this study, using RNA sequencing technology, we analysed the expression profiles of mRNAs in the cochlear tissues of ARHL‐susceptible C57BL/6J (C57) mice and relatively resistant CBA/CaJ (CBA) mice. We found that the mRNA related to stress granules (SGs) was significantly increased in the cochlear tissues of ARHL mice. Combined with current research on the pyroptosis of cochlear hair cells, we constructed an aging model of House Ear Institute‐Organ of Corti 1 (HEI‐OC1) in vitro and found that the expression of SG was upregulated and the expression of pyroptosis‐related proteins showed a synergistic upregulation. Furthermore, functional studies involving both G3BP1 knockdown and overexpression suggested that stress granules exert a protective effect by negatively regulating pyroptosis in HEI‐OC1 cells. In summary, the results of this study indicate that with aging, the formation of SG increases and the activation of pyroptosis‐related markers is enhanced. The dysfunction of G3BP1 further exacerbates these pyroptosis markers, supporting the protective role of SG homeostasis in inhibiting pyroptosis activation under stress conditions. Preliminary verification suggests that the SG‐pyroptosis axis may be a potential target for regulating the pyroptosis of cochlear hair cells.

## 1 Introduction

ARHL is a degenerative process of the aging auditory system, characterised by progressive, bilateral and symmetric sensorineural hearing impairment [1]. Hearing loss can compromise social functioning and communication, adversely affect interpersonal relationships and mental health and markedly reduce quality of life [2]. Given the substantial socioeconomic burden imposed by ARHL, there is a pressing need to develop therapeutic interventions and a deeper understanding of the underlying molecular mechanisms [3].

Studies have identified that the pathological basis of ARHL mainly includes the degeneration of hair cells (HC), atrophy of the stria vascularis (SV) and loss of neurons jointly [4]. Under homeostatic conditions, the organ of Corti is relatively immune‐quiescent, supported by the blood–labyrinth barrier and local immune regulation [5]. With aging, however, cochlear tissues exhibit features consistent with chronic, low‐grade inflammation (inflammaging). Damage‐associated signals released from stressed cochlear cells activate resident macrophages and non‐immune stromal populations, amplifying pro‐inflammatory pathways and increasing local cytokine/chemokine production [6]. Such sustained inflammatory pressure can reduce hair‐cell stress tolerance by disrupting redox balance, calcium handling and mitochondrial function, thereby accelerating sensory‐cell dysfunction and loss [7].

Pyroptosis has been observed in other neurodegenerative diseases as well. It is a lytic form of cell death canonically linked to inflammasome activation and characterised by caspase‐1–dependent processing of inflammatory cytokines and cleavage of gasdermin D (GSDMD), whose N‐terminal fragment forms membrane pores and promotes cellular swelling and rupture [8]. In the cochlea, NLRP3 signalling has been implicated in response to diverse stressors that are relevant to aging, including oxidative stress, metabolic perturbation and sterile inflammation [9]. Several studies have reported increased expression or activation of pyroptosis‐associated components (e.g., NLRP3, cleaved caspase‐1 and GSDMD‐N) in models of cochlear aging and degeneration, and pharmacologic or genetic modulation of upstream inflammatory pathways can sometimes mitigate cochlear pathology [10, 11]. At the same time, important questions remain regarding the timing, cellular sources and causal contribution of pyroptosis in ARHL. Moreover, whether inflammasome/pyroptosis activation is an initiating event in hair‐cell degeneration or a secondary amplification mechanism downstream of tissue stress is still debated. Clarifying how pyroptosis is regulated within the aging cochlear microenvironment—and identifying actionable molecular nodes that connect chronic stress to inflammasome activation—would advance mechanistic understanding and therapeutic design.

SGs are intracellular ‘granules’ formed under diverse stress conditions and are, in essence, dynamic condensates composed of RNA and proteins [12]. Upon cellular stress, phosphorylation of eIF2α inhibits translation initiation, increasing the pool of non‐translating mRNAs. These mRNAs associate with RNA‐binding proteins such as G3BP1 and TIA‐1 and undergo liquid–liquid phase separation (LLPS) to assemble SG [13]. Once the stress is relieved, SGs rapidly disassemble, thereby preserving transcript integrity and enabling translational reprogramming to facilitate stress adaptation [14]. SGs represent one plausible regulatory layer bridging cellular stress adaptation and inflammatory signalling. The relationship between SGs and inflammasome pathways is increasingly appreciated as context‐dependent: SGs assembly can dampen certain stress‐activated signalling cascades, yet prolonged or dysregulated SG dynamics may also contribute to maladaptive inflammation [15]. However, whether a G3BP1–SG axis is engaged in ARHL, and how it relates to pyroptosis‐associated programs in the cochlea, remains insufficiently defined.

Here, we aimed to test the hypothesis that SG‐related responses, centred on G3BP1, are transcriptionally coupled to pyroptosis‐associated activation in ARHL‐relevant conditions. We performed cochlear transcriptomic profiling using mouse strains that are susceptible or relatively resistant to ARHL to identify coordinated changes in SG/translation‐stress signatures and pyroptosis‐related gene programs, and prioritised candidate nodes based on differential expression and association analyses. Key pyroptosis effectors were then validated at the protein level by assessing cleaved caspase‐1 and the GSDMD‐N. To investigate the functional role of G3BP1, we performed reciprocal loss‐ and gain‐of‐function experiments in HEI‐OC1 cells and assessed caspase‐1/GSDMD‐associated signalling and inflammatory cytokine release. Our study aims to define a mechanistically informative link between the G3BP1–SG axis and inflammatory cell death, thereby connecting chronic cochlear stress to ARHL pathogenesis.

## 2 Method and Materials

### 2.1 Animal Model

Five 9‐month‐old male C57BL/6J and five age‐matched male CBA/CaJ mice were purchased from Sibeifu (Beijing) Biotechnology Co. Ltd. All animals were maintained under specific pathogen‐free (SPF) conditions and acclimated for 1 week prior to experimentation. All procedures were approved by the Ethics Committee of Peking Union Medical College Hospital (approval No. XHDW‐2025‐033).

### 2.2 Auditory Brainstem Responses(ABR)

Mice were anaesthetised by inhalation and placed on a thermostatically controlled heating pad for ABR recordings in a sound‐attenuating chamber. Subdermal needle electrodes were positioned beneath the pinna of the test ear (negative), at the vertex (recording) and at the ear tip (positive), with the ground electrode placed over the muscle of the contralateral hind limb [16]. ABRs were acquired using a TDT system with SigGen/BioSig software. Tone‐burst stimuli at 8, 16, 24 and 32 kHz were presented starting at 90 dB SPL and decreased in 5‐dB steps to determine the minimum intensity that elicited a response. The ABR threshold was defined as the lowest stimulus intensity that produced a reliable and reproducible waveform in at least two independent trials.

### 2.3 Cochlear RNA Sequencing and Differential Expression Analysis

RNA concentration/purity was measured by NanoDrop and integrity was assessed by RNA agarose gel electrophoresis. Poly(A) + mRNA was purified, fragmented (~200–300 bp) and reverse‐transcribed to generate double‐stranded cDNA, followed by end repair, A‐tailing and adapter ligation. Libraries were bead‐purified/size‐selected (final size ~300–400 bp), PCR‐amplified, quantified by qRT‐PCR, pooled by index, denatured and sequenced by synthesis. Raw reads were filtered to obtain clean reads and aligned to the reference genome to evaluate mapping quality, saturation and coverage. Gene expression was quantified, and differential expression between groups was determined, followed by visualisation and Gene Ontology (GO) and KEGG pathway functional enrichment analyses [17].

### 2.4 Cell Culture and H_2_O_2_ ‐Induced Senescence Model

HEI‐OC1 cells were purchased from iCell Bioscience (China) and cultured in high‐glucose DMEM (Gibco, USA) supplemented with 10% foetal bovine serum (FBS; Gibco, USA) at 37°C under 5% CO_2_. An oxidative stress–induced senescence model was established using hydrogen peroxide (H_2_O_2_). HEI‐OC1 cells were seeded and allowed to adhere overnight [18]. The next day, the medium was replaced with basal culture medium containing H_2_O_2_ at final concentrations of 50 μM. Cells were continuously exposed to 50 μM H_2_O_2_ for 36 or 72 h without a subsequent recovery period.

### 2.5 Senescence‐Associated β‐Galactosidase Stain

HEI‐OC1 cells were assessed for senescence‐associated β‐galactosidase (SA‐β‐gal) activity using a commercial β‐galactosidase staining kit (Beyotime, Shanghai, China) following the manufacturer's instructions.

### 2.6 Western Blotting

Cochlear tissues or HEI‐OC1 cells were homogenised in ice‐cold RIPA lysis buffer containing protease and phosphatase inhibitors. After centrifugation at 12,000×*g* for 15 min at 4°C to remove cellular debris, the supernatants were collected and total protein concentrations were measured with a BCA Protein Assay Kit (Abbkine, China). Equivalent quantities of protein were loaded onto 4%–20% ACE gradient gels for SDS–PAGE electrophoresis, followed by electrotransfer onto PVDF membranes. The membranes were then blocked with 5% non‐fat milk in TBST for 1 h at ambient temperature, and subsequently probed overnight at 4°C with the following primary antibodies: G3BP1 (Proteintech, China), cleaved caspase‐1 (Immunoway, China), GSDMD‐N (Immunoway, China), β‐actin (Proteintech, China) and GAPDH (Proteintech, China). Following three washes with TBST, the membranes were incubated with HRP‐linked secondary antibodies for 1 h at room temperature. Immunoreactive bands were detected using enhanced chemiluminescence (ECL) substrate and captured with a chemiluminescence imaging system [19]. Densitometric analysis of protein bands was performed with ImageJ software, with β‐actin and GAPDH serving as the loading control for normalisation.

### 2.7 Quantitative Real‐Time PCR (qRT‐PCR)

Total RNA was extracted from cochlear tissues or HEI‐OC1 cells using a column‐based RNA extraction kit (Beyotime, China) according to the manufacturer's instructions. RNA concentration and purity were measured by NanoDrop. cDNA was synthesised from equal amounts of total RNA using the PrimeScript RT Reagent Kit (Takara, Japan; RR047A). Quantitative real‐time PCR was performed using TB Green Premix Ex Taq II (Takara, Japan; RR820A/B) on a real‐time PCR system under the manufacturer‐recommended cycling conditions. Relative mRNA expression levels were normalised to the internal control gene (e.g., GAPDH) and calculated using the 2^−ΔΔCt^ method. Primer sequences are listed in Supplementary Table 1.

### 2.8 Immunofluorescence Staining

HEI‐OC1 cells were seeded onto sterile glass coverslips placed in six‐well plates and allowed to adhere overnight. After the indicated treatments, cells were fixed with 4% paraformaldehyde for 15–20 min at room temperature, permeabilized with 0.3% Triton X‐100 in PBS for 10–15 min and blocked with 5% normal goat serum in PBS for 1 h at room temperature. Cells were then incubated overnight at 4°C with primary antibodies against G3BP1 (Proteintech), cleaved caspase‐1 (Immunoway), GSDMD‐N (Immunoway) and ASC (AdipoGen), as indicated for each experiment. After washing with PBS, coverslips were incubated with fluorophore‐conjugated secondary antibodies (Proteintech) for 1 h at room temperature in the dark. Nuclei were counterstained with DAPI, and coverslips were mounted using an anti‐fade mounting medium. Fluorescence images were acquired using a fluorescence or confocal microscope under identical exposure settings between groups and analysed using ImageJ.

### 2.9 ELISA Measurement of IL‐1β and IL‐18 in HEI‐OC1 Cell Culture Supernatants

ELISA was performed using commercial kits from MultiSciences (Lianke Biotech, Hangzhou, China) according to the manufacturer's instructions. Briefly, standards and samples were added to the antibody‐coated plates, followed by incubation with the detection antibody. After washing, Streptavidin‐HRP was added and incubated. The plates were then washed and developed with TMB substrate, and the reaction was terminated with stop solution. Absorbance was measured at 450 nm using a microplate reader, and protein concentrations were calculated from the standard curve.

### 2.10 siRNA‐Mediated G3BP1 Knockdown and Plasmid‐Mediated G3BP1 Overexpression in HEI‐OC1 Cells

HEI‐OC1 cells were seeded in six‐well plates and allowed to attach overnight. For G3BP1 knockdown, cells were transfected with G3BP1‐specific siRNA or negative control siRNA (siNC; Genomeditech, China) using Doornor transfection reagent according to the manufacturer's instructions. The siRNA stock solution (20 μM) was diluted to a final working concentration of 50 nM. Three G3BP1‐targeting siRNAs were initially screened by qRT‐PCR, and the siRNA showing the highest knockdown efficiency was selected for subsequent experiments. Knockdown efficiency was further confirmed by western blotting.

For G3BP1 overexpression, cells were transfected with a G3BP1 overexpression plasmid or the corresponding empty vector using Lipofectamine 3000 (Invitrogen, USA) according to the manufacturer's instructions. Successful G3BP1 overexpression was confirmed by western blotting using an anti‐FLAG antibody.

At 24 h after transfection, cells were exposed to 50 μM H_2_O_2_ for 36 or 72 h. The expression of pyroptosis‐associated proteins, including GSDMD‐N and cleaved caspase‐1, was subsequently assessed by western blotting. In the overexpression experiments, IL‐1β and IL‐18 levels in the culture supernatants were additionally measured by ELISA.

### 2.11 Statistical Analysis

All data are presented as mean ± standard error of the mean (SEM), except for the auditory brainstem response (ABR) threshold data in Table 1 and Figure‐1B, which are presented as mean ± standard deviation (SD). All experiments included at least three independent experiments or biological replicates, unless otherwise specified. Statistical analyses were performed using GraphPad Prism (10, GraphPad Software, USA). For comparisons between two groups, an unpaired two‐tailed Student's *t*‐test was used. For comparisons among multiple groups, one‐way or two‐way analysis of variance (ANOVA) followed by appropriate post hoc multiple‐comparison tests (e.g., Tukey's test) was applied, as indicated in the figure legends. For ABR threshold analysis across frequencies, two‐way ANOVA was used to assess the effects of group and frequency. A value of *p* < 0.05 was considered statistically significant.

## 3 Results

### 3.1 Distinct Hearing Phenotypes and Strain‐Associated Cochlear Transcriptomic Signatures in Aged C57BL/6J and CBA/CaJ Mice

We performed ABR testing in 9‐month‐old C57 and CBA mice at 8, 16, 24 and 32 kHz. Compared with aged CBA mice, C57 mice exhibited significantly elevated ABR thresholds at all tested frequencies (Figure 1 and Table 1). Based on this functional difference, cochlear tissues from both groups were subsequently subjected to transcriptomic (RNA‐seq) profiling to delineate molecular signatures associated with susceptibility to ARHL.

**FIGURE 1:**
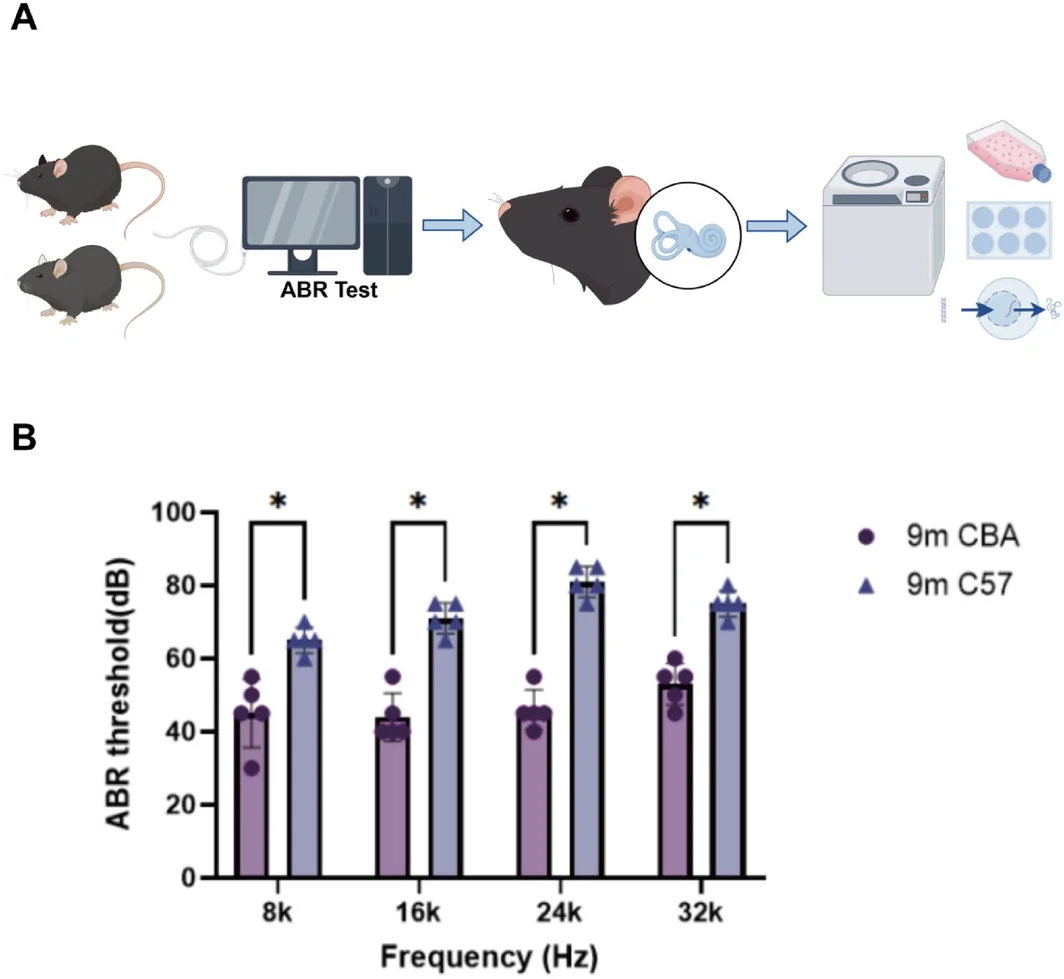
(A) Experimental flowchart. After ABR testing was conducted on C57 and CBA mice, the cochleae were sampled and RNA‐seq was performed. The results were analysed, followed by in vitro protein level verification and functional verification. (B) ABR hearing test results of the two groups of 9‐month‐old mice. The hearing threshold of C57 mice was significantly higher than that of CBA.

**TABLE 1:** ABR thresholds in mice.

ABR threshold (dB SPL)	8 kHz	16 kHz	24 kHz	32 kHz
9mCBA (*n* = 5)	45 ± 9.35	44 ± 6.52	46 ± 5.48	53 ± 5.70
9mC57 (*n* = 5)	65 ± 3.54	71 ± 4.18	81 ± 4.18	75 ± 3.54
*p*	0.002	< 0.001	< 0.001	< 0.001

After RNA sequencing of cochlear tissues from 9‐month‐old C57 and CBA mice, we first assessed global transcriptomic differences. Principal component analysis (PCA) revealed a clear separation between the two groups at the transcriptome level (Figure 2A). Using the predefined criteria for differential expression (|log2FC| ≥ 0.58, FDR < 0.05), we identified 3439 upregulated and 3080 downregulated genes in C57 mice relative to CBA mice. These results are presented in the volcano plot in Figure 2B. GO and KEGG pathway analysis showed that DEGs were significantly enriched in ribosome‐ and translation‐related processes, including ribosomal subunit assembly, as well as inflammatory and immune‐related pathways, such as NF‐κB signalling and complement and coagulation cascades (Figure 2C,E). GSEA further revealed enrichment of homeostasis‐related gene sets, including chemical homeostasis, general homeostatic processes and monocarboxylic acid metabolism (Figure 2D), indicating that the distinct hearing phenotypes of the two strains were accompanied by alterations in cochlear cellular homeostasis.

**FIGURE 2:**
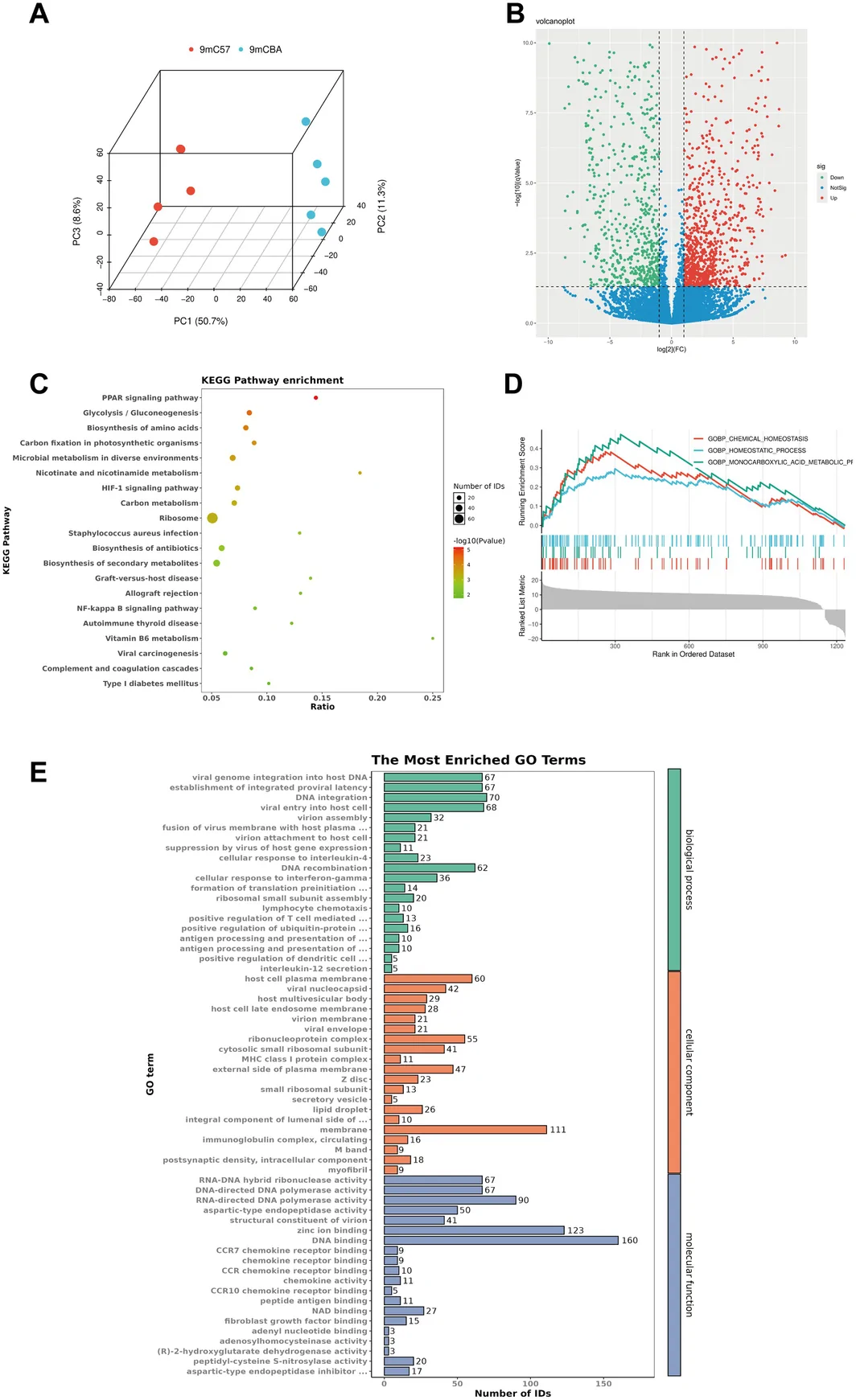
Transcriptome sequencing analysis results. (A) The PCA graph shows a significant separation between the two groups at the transcriptome level. (B) The volcano plot displays genes with significantly upregulated and downregulated expressions, with 3439 and 3080 genes respectively. (E) and (C) are the results of GO and KEGG enrichment analysis. (D) GSEA analysed the enrichment of homeostasis‐related programs, including chemical/homeostasis processes and monocarboxylic acid metabolism, and performed positive enrichment.

Given the enrichment of RNA metabolism, ribosome and cellular stress‐related processes, we further focused on G3BP1, a core regulator of stress granule assembly, for subsequent protein validation and functional investigation. RT‐qPCR and Western blot analyses showed no significant difference in cochlear G3BP1 expression between 2‐month‐old CBA and C57 mice. In contrast, G3BP1 protein levels were significantly higher in 9‐month‐old C57 mice than in age‐matched CBA mice (Figure 3), suggesting that the strain‐associated difference in G3BP1 expression becomes apparent with aging.

**FIGURE 3:**
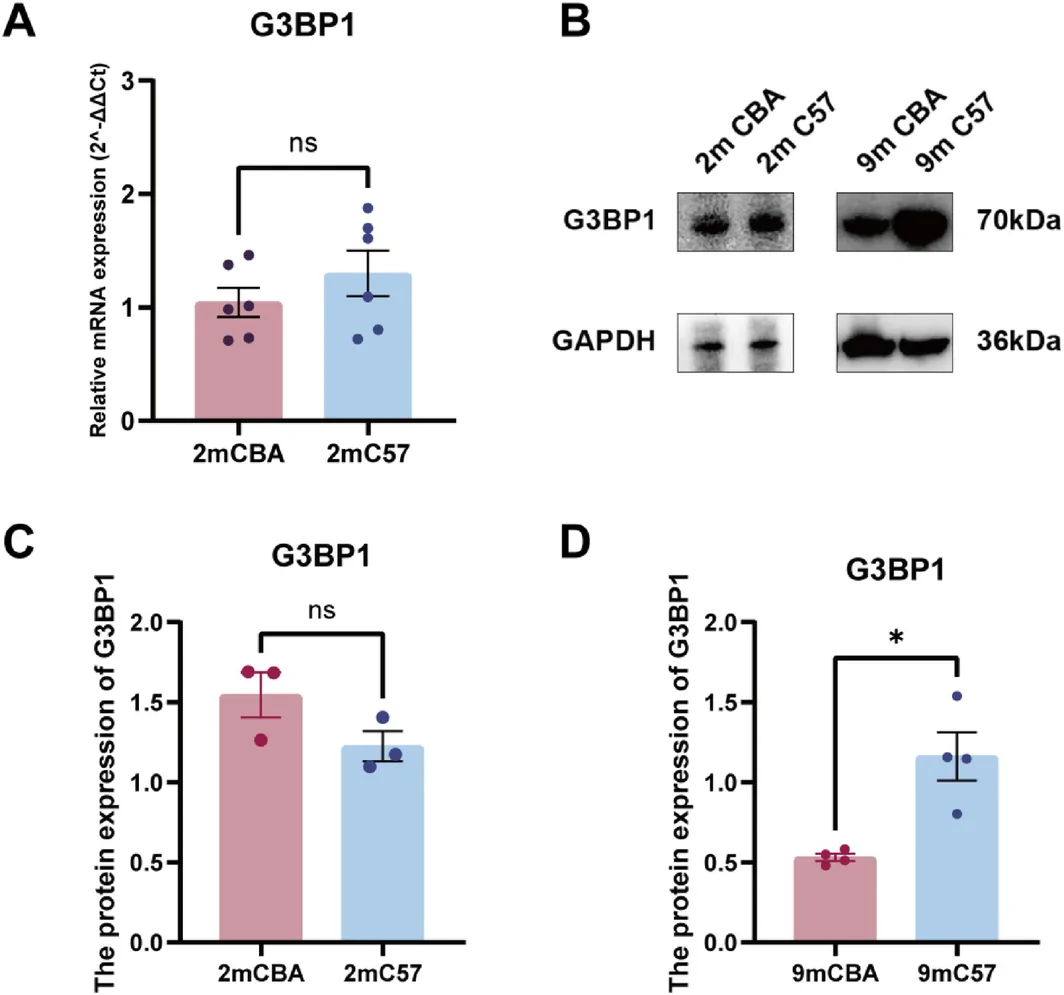
Comparison of cochlear G3BP1 expression between CBA/CaJ and C57BL/6J mice at 2 and 9 months of age. (A) No significant difference was observed between the two strains in RT‐qPCR analysis of G3BP1 mRNA expression in cochlear tissues (*n* = 6 per group). (B) Representative Western blot images of G3BP1 protein expression in cochlear tissues from 2‐month‐old and 9‐month‐old CBA and C57 mice. (C, D) Densitometric quantification of G3BP1 protein levels normalised to GAPDH in 2‐month‐old (C) and 9‐month‐old (D) mice. No significant difference was observed between the two strains at 2 months of age, whereas G3BP1 protein levels were significantly higher in 9‐month‐old C57 mice than in age‐matched CBA mice. Data are presented as the mean ± SEM. Each dot represents one biological replicate. ns, not significant; *p < 0.05.

### 3.2 Stress Granule and Pyroptosis‐Associated Changes in the H_2_O_2_ ‐Induced HEI‐OC1 Model

Treatment with 50 μM H_2_O_2_ induced a senescence‐like phenotype in HEI‐OC1 cells, as evidenced by an increased proportion of SA‐β‐gal‐positive cells and elevated p21 protein expression at both 36 and 72 h (Figure 4A–C,F). The mRNA levels of the SASP‐related factors IL‐6 and TNF‐α showed no significant changes at 36 h but were significantly increased at 72 h (Figure 4D,E), further supporting the development of a senescence‐associated phenotype under prolonged oxidative stress. Meanwhile, G3BP1 expression was significantly increased following H_2_O_2_ treatment at both time points (Figure 4G,H). The pyroptosis‐associated markers GSDMD‐N and cleaved caspase‐1 were also elevated after H_2_O_2_ exposure, with cleaved caspase‐1 significantly increased at both 36 and 72 h and GSDMD‐N significantly increased at 72 h (Figure 4G–J). Together, these results indicate that H_2_O_2_‐induced senescence‐like changes are accompanied by increased G3BP1 expression and activation of caspase‐1/GSDMD‐associated signalling in HEI‐OC1 cells.

**FIGURE 4:**
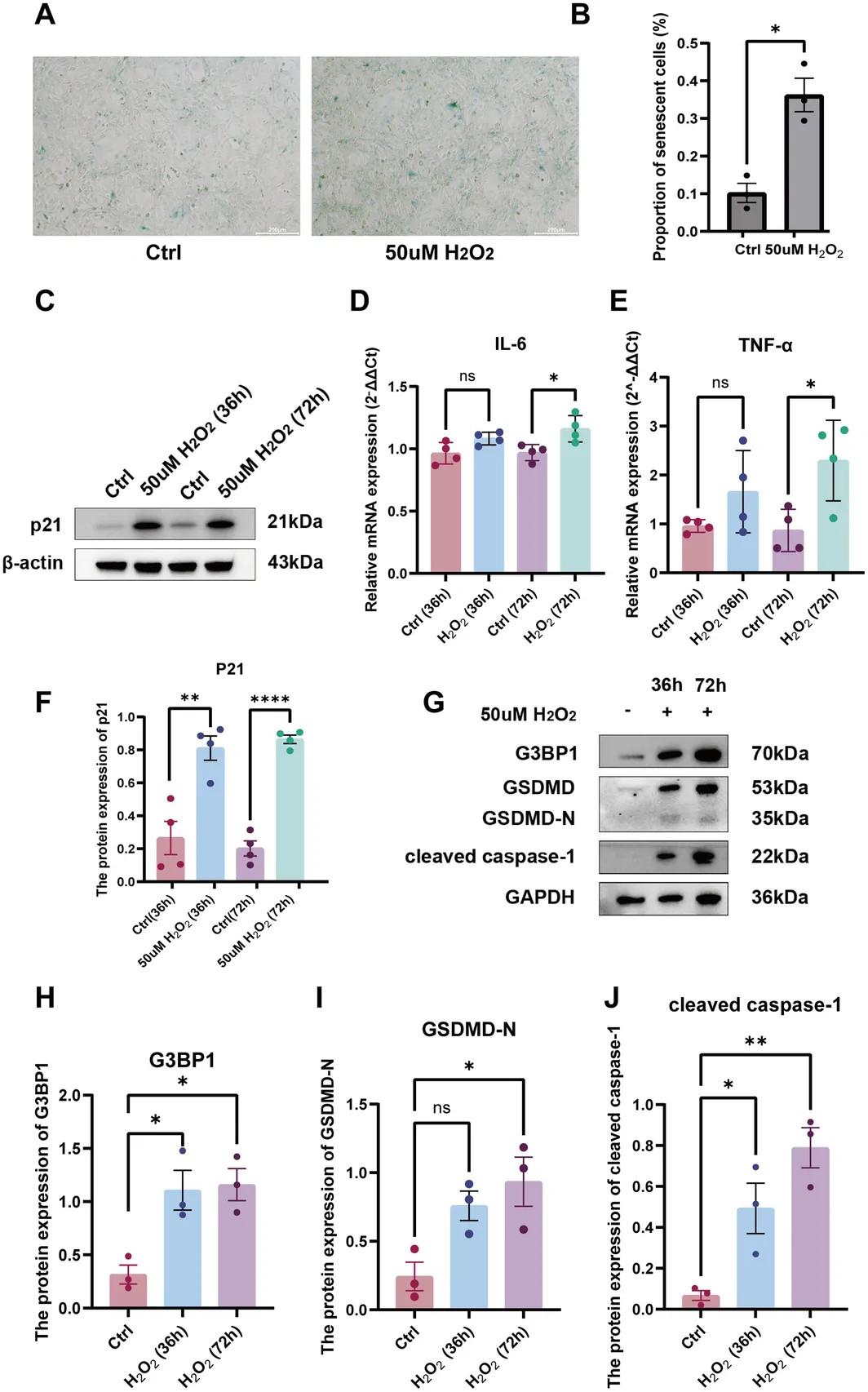
H_2_O_2_ induces a senescence‐associated phenotype and pyroptosis‐associated signalling in HEI‐OC1 cells. (A) Representative SA‐β‐gal staining images of control and 50 μM H_2_O_2_‐treated HEI‐OC1 cells. (B) Quantification of SA‐β‐gal‐positive senescent cells. (C) Representative Western blot images of p21 expression after 50 μM H_2_O_2_ exposure for 36 and 72 h. β‐actin was used as the loading control. (D, E) Relative mRNA expression levels of the senescence‐associated secretory phenotype (SASP)‐related factors IL‐6 and TNF‐α at 36 and 72 h, as determined by qRT‐PCR. (F) Quantification of p21 protein expression. (G) Representative Western blot images of G3BP1, GSDMD/GSDMD‐N and cleaved caspase‐1 after H_2_O_2_ exposure for 36 and 72 h. GAPDH was used as the loading control. (H–J) Quantification of G3BP1, GSDMD‐N and cleaved caspase‐1 protein levels, respectively.

### 3.3 Functional Modulation of G3BP1 Reveals Its Protective Role in Pyroptosis‐Associated Signalling in HEI‐OC1 Cells

Among the three candidate siRNAs, siRNA‐2 showed the highest knockdown efficiency by qRT‐PCR and was therefore selected for subsequent experiments (Figure 5). Effective G3BP1 knockdown was further confirmed by Western blotting in the functional experiments (Figure 6A–C,G). To investigate the effects of G3BP1 silencing under aging conditions, we measured at two time points, following 36 and 72 h H_2_O_2_ exposure after transfection. HEI‐OC1 cells were divided into four groups: control group, H_2_O_2_‐induced model group, siNC + H_2_O_2_ group and siG3BP1 (siRNA‐2) + H_2_O_2_ group. Notably, under H_2_O_2_ treatment, G3BP1 knockdown further increased the levels of cleaved caspase‐1 and GSDMD‐N, indicating exacerbated caspase‐1/GSDMD‐mediated pyroptotic signalling (Figure 6A,B,D,E,H,I). In addition, p21 expression was further increased following G3BP1 knockdown at 36 h, whereas no significant difference was observed at 72 h (Figure 6F,J).

**FIGURE 5:**
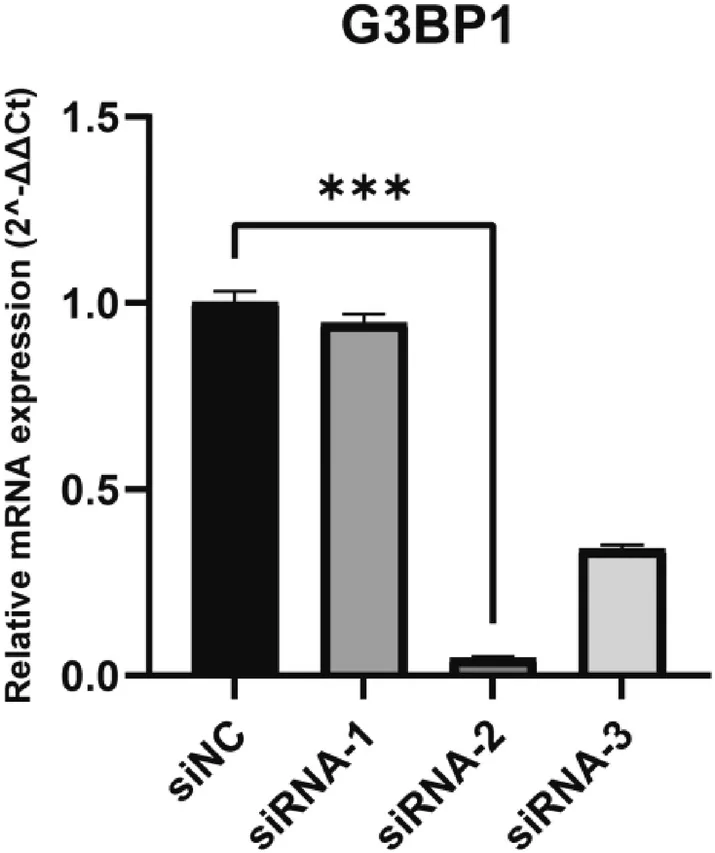
Screening of siRNAs targeting G3BP1 in HEI‐OC1 cells. siRNA‐2 showed the strongest knockdown efficiency and was therefore selected for subsequent experiments. Data are presented as mean ± SEM. ***p < 0.001.

**FIGURE 6:**
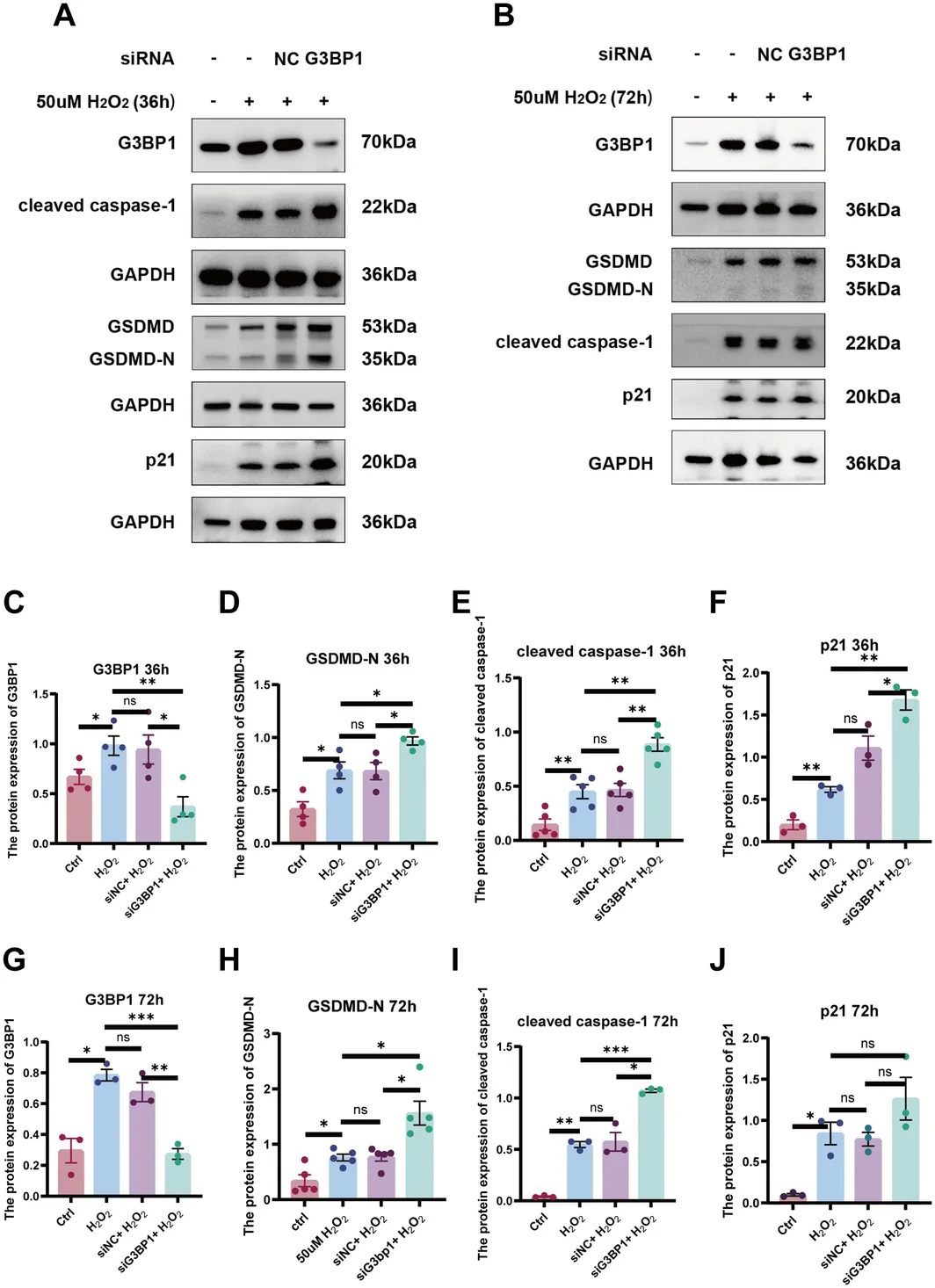
G3BP1 knockdown enhances H_2_O_2_‐induced pyroptosis‐associated activation in HEI‐OC1 cells. (A, B) WB showed the protein levels of G3BP1, pyroptosis‐related proteins (GSDMD‐N, cleaved caspase‐1) and aging protein p21 in the four groups (control group, H_2_O_2_ model group, siNC +H_2_O_2_ model group and siG3BP1 +H_2_O_2_ model group) after 36 and 72 h of H_2_O_2_ exposure. (C–F) were the analysis of the grey values of the protein bands of G3BP1, GSDMD‐N, cleaved caspase‐1 and p21 at 36 h. (G–J) Corresponding quantification at 72 h. ns, not significant (*p* ≥ 0.05); *p < 0.05; **p < 0.01; ***p < 0.001; ****p < 0.0001.

To further validate the role of G3BP1, a G3BP1 overexpression plasmid was constructed (Figure 7A), and successful overexpression was confirmed by robust FLAG expression at both 36 and 72 h (Figure 7B,G). Compared with the vector control, G3BP1 overexpression showed a decreasing trend in GSDMD‐N and cleaved caspase‐1 levels at 36 h, although the differences did not reach statistical significance (Figure 7C,D). At 72 h, however, G3BP1 overexpression significantly reduced both GSDMD‐N and cleaved caspase‐1 levels (Figure 7E,F). Consistently, ELISA analysis showed that G3BP1 overexpression significantly decreased IL‐18 levels at both time points, while IL‐1β levels were not significantly altered (Figure 7H,I). Immunofluorescence analysis further showed increased G3BP1 fluorescence following overexpression, reaching statistical significance at 72 h, whereas ASC fluorescence intensity showed a clear tendency towards reduction at both time points, although the differences did not reach statistical significance (Figure 8, Figure 9A,B).

**FIGURE 7:**
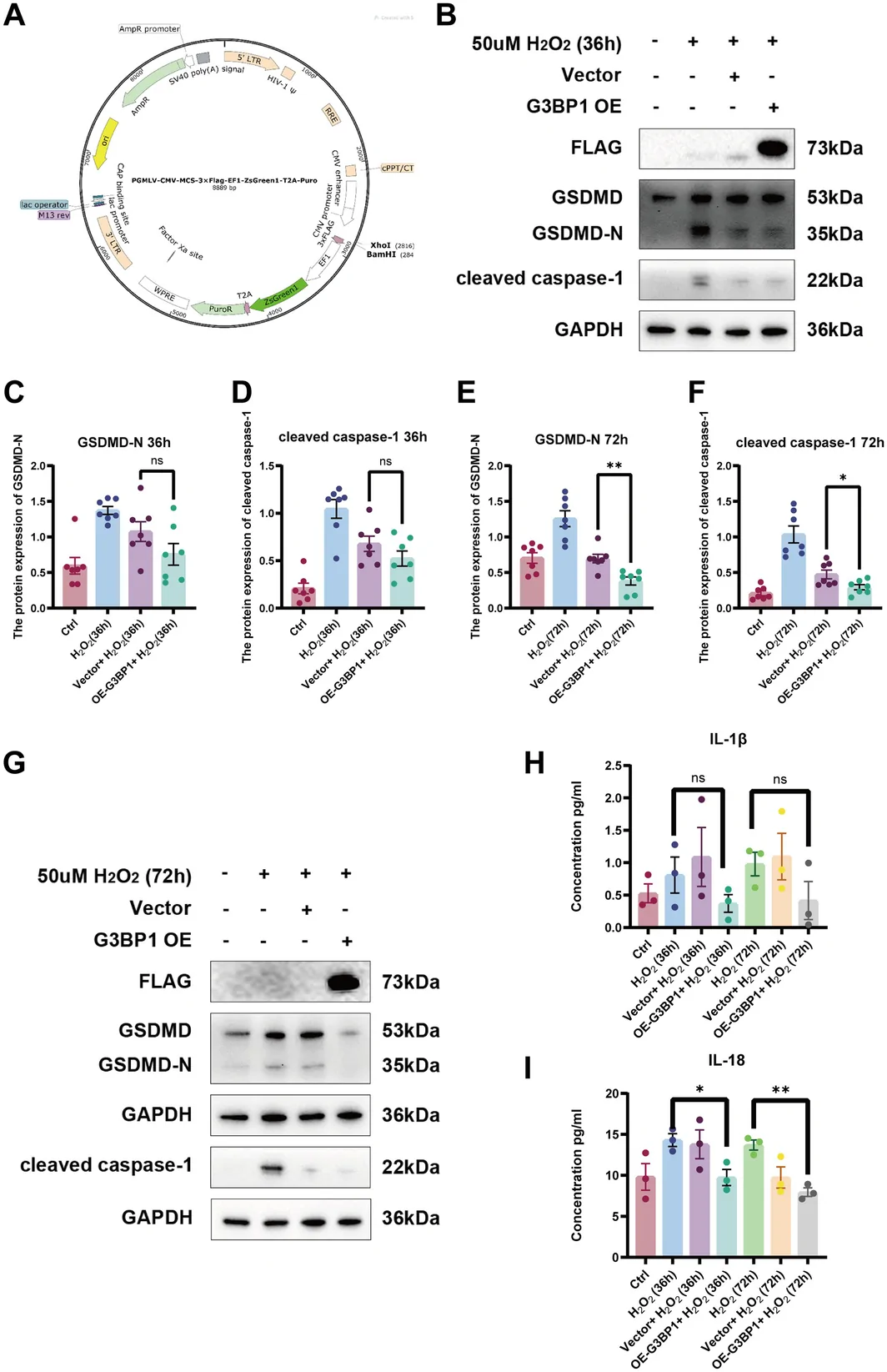
(A) Schematic representation of the G3BP1 overexpression plasmid construct. (B) Representative Western blot images of FLAG, GSDMD/GSDMD‐N and cleaved caspase‐1 in HEI‐OC1 cells exposed to 50 μM H_2_O_2_ for 36 h following G3BP1 overexpression. (C, D) Quantification of GSDMD‐N and cleaved caspase‐1 protein levels at 36 h. (E, F) Quantification of GSDMD‐N and cleaved caspase‐1 protein levels at 72 h. (G) Representative Western blot images of FLAG, GSDMD/GSDMD‐N and cleaved caspase‐1 after 72 h of H_2_O_2_ exposure. GAPDH was used as the loading control. (H, I) Concentrations of IL‐1β and IL‐18 in the indicated groups at 36 and 72 h. Data are presented as mean ± SEM. ns, not significant (*p* ≥ 0.05); *p < 0.05; **p < 0.01.

**FIGURE 8:**
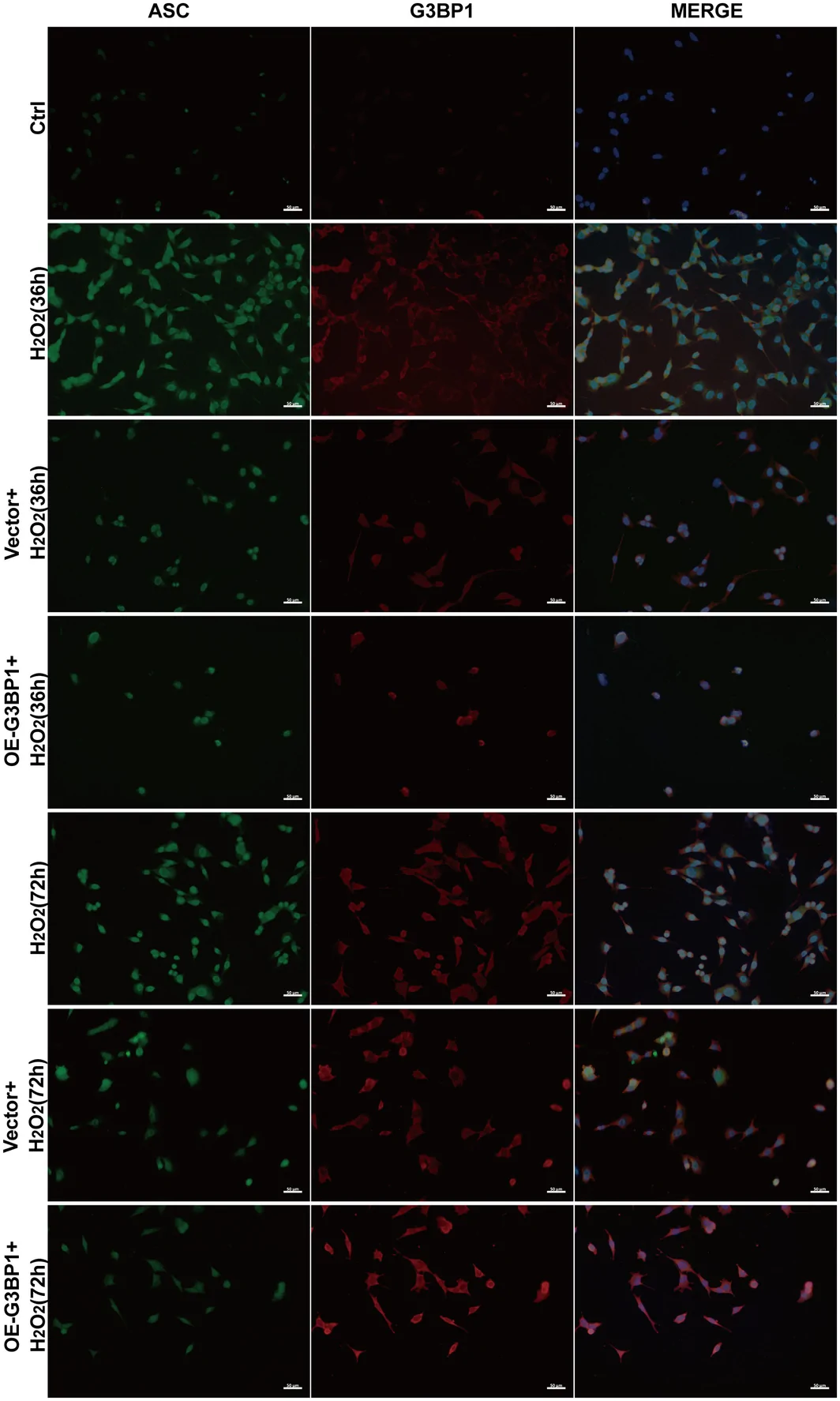
Effects of G3BP1 overexpression on ASC and G3BP1 expression in H_2_O_2_‐treated HEI‐OC1 cells. Representative immunofluorescence images showing ASC (green), G3BP1 (red) and nuclei (DAPI, blue) in control cells and HEI‐OC1 cells exposed to 50 μM H_2_O_2_ for 36 or 72 h, with or without vector or G3BP1 overexpression. Merge images show the spatial distribution of ASC and G3BP1 signals. Scale bar = 20 μm.

**FIGURE 9:**
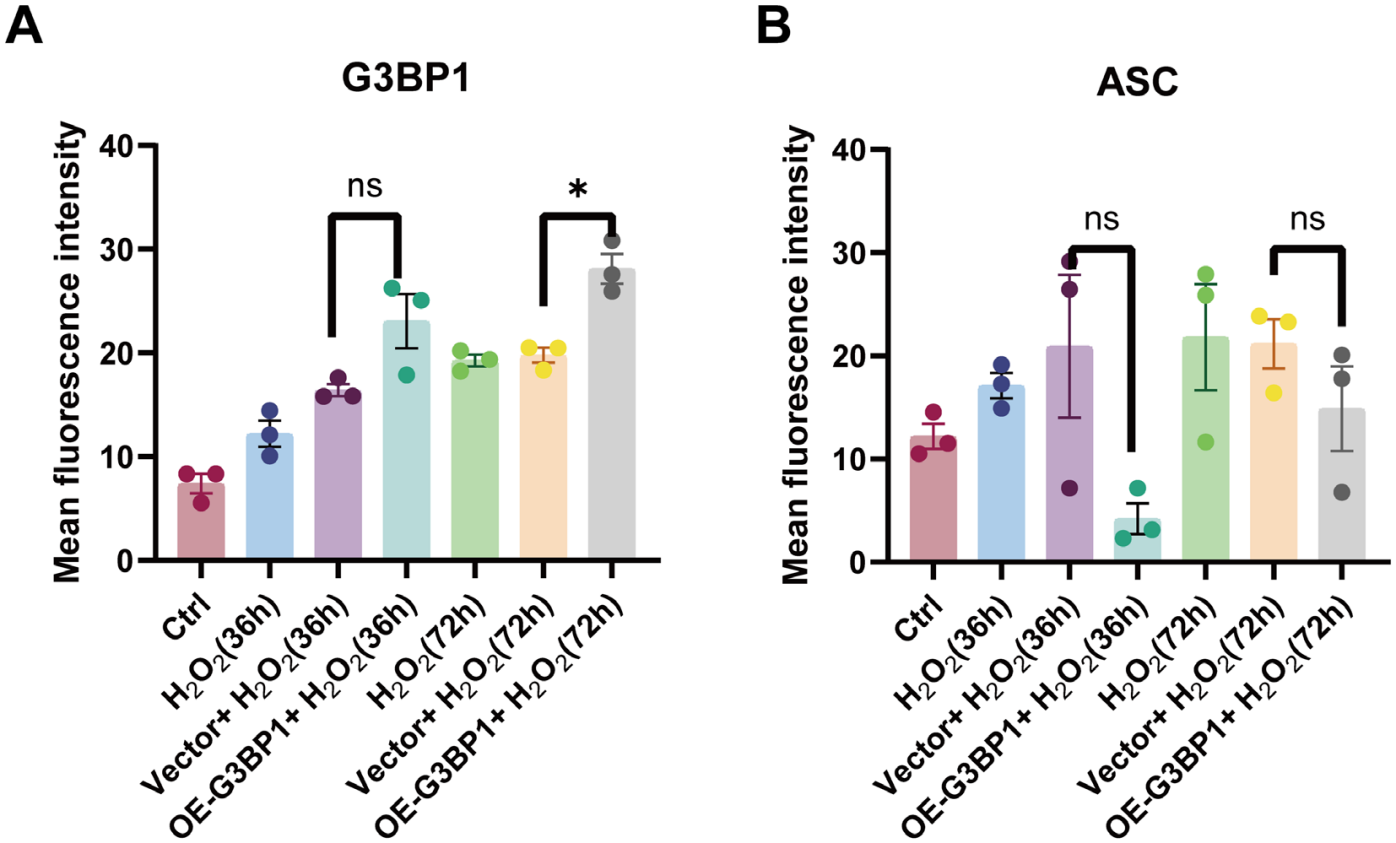
Quantification of G3BP1 and ASC fluorescence intensity following G3BP1 overexpression in H_2_O_2_‐treated HEI‐OC1 cells. (A) Quantification of mean G3BP1 fluorescence intensity in control cells and cells exposed to 50 μM H_2_O_2_ for 36 or 72 h, with or without vector or G3BP1 overexpression. (B) Quantification of mean ASC fluorescence intensity in the corresponding groups. Data are presented as mean ± SEM. ns, not significant; *p < 0.05.

Collectively, these bidirectional findings support a protective role of G3BP1 against H_2_O_2_‐induced pyroptosis‐associated activation in HEI‐OC1 cells.

## 4 Discussion

This study started with transcriptomic analysis of cochlear tissue from mouse strains with different susceptibility to ARHL. SG‐related biological processes were enriched in the strain more vulnerable to hearing loss, and G3BP1 was identified as a candidate molecule for subsequent investigation. Because the original RNA‐sequencing analysis compared 9‐month‐old C57 and CBA mice, strain‐specific genetic effects cannot be fully separated from aging‐related changes. To partially address this issue, we examined G3BP1 expression in young and aged mice of both strains. G3BP1 levels were comparable between young C57 and CBA mice, whereas a significant difference emerged in aged animals, suggesting that this difference is unlikely to be purely constitutive and may develop with strain‐dependent susceptibility to hearing loss. Nevertheless, the transcriptomic findings should be interpreted primarily as susceptibility‐associated differences rather than direct aging‐induced changes. Consistent with the importance of post‐transcriptional stress regulation in cochlear aging, recent work identified the RNA‐binding protein HuR as an age‐dependent regulator of hair‐cell maintenance, with HuR overexpression alleviating ARHL and HuR deficiency accelerating auditory dysfunction in mice [20]. Moreover, recent single‐nucleus transcriptomic profiling has demonstrated substantial cellular and molecular heterogeneity within the cochlea [21], emphasising that bulk cochlear transcriptomic changes cannot be assigned to specific cell populations without further cell‐type‐resolved analysis.

To further investigate these findings, an H_2_O_2_‐induced senescence‐associated model was established in HEI‐OC1 cells. H_2_O_2_ exposure increased SA‐β‐gal positivity and p21 expression, while the SASP‐related factors IL‐6 and TNF‐α were significantly elevated at 72 h. In parallel, G3BP1 expression increased together with enhanced caspase‐1/GSDMD‐associated signalling. These findings suggest that prolonged oxidative stress is accompanied by a senescence‐associated phenotype and increased pyroptosis‐associated activation.

G3BP1 was selected as a candidate regulatory factor for further validation. G3BP1 knockdown enhanced GSDMD‐N and cleaved caspase‐1 expression, whereas G3BP1 overexpression produced the opposite effect. IL‐18 release was also reduced at both 36 and 72 h, whereas IL‐1β was not significantly altered. Together, these findings support a G3BP1‐dependent protective effect against H_2_O_2_‐induced pyroptosis‐associated activation. However, because G3BP1 also has biological functions beyond SG assembly, the present results do not establish that this effect is exclusively SG‐dependent.

SGs are widely regarded as an adaptive stress‐response mechanism that preserves mRNA integrity and supports translational reprogramming [22]. Increasing evidence indicates that SG functions are context‐ and time‐dependent: transient SG assembly often plays a protective role, whereas prolonged or dysregulated SGs dynamics may become less beneficial as stress endures [23, 24, 25, 26]. This distinction is particularly relevant to the aging cochlea, where chronic oxidative/metabolic stress and low‐grade inflammation impose sustained proteostasis pressure. Consistently, single‐cell profiling of aging primate cochleae has revealed progressive sensory hair‐cell loss, increased neuroinflammatory changes in spiral ganglion neurons and marked stria vascularis atrophy, highlighting the multicellular and heterogeneous nature of cochlear aging [27]. Our findings suggest that increased G3BP1 may represent a compensatory stress response that limits inflammatory signalling, although whether this protection persists during long‐term aging remains to be determined.

Pyroptosis is an inflammatory form of programmed cell death characterised by the propagation of inflammatory signalling, pore formation in the plasma membrane and eventual cell lysis following caspase‐1 activation and GSDMD cleavage [28]. In recent years, this pathway has drawn increasing attention in degenerative and stress‐related disorders. In the cochlea, accumulating evidence suggests that aging is accompanied by persistent low‐grade inflammation and oxidative stress [29]. Within this context, pyroptosis may represent one mechanism by which hair‐cell injury promotes local inflammatory amplification and increases the vulnerability of surrounding tissues [30]. Recent evidence demonstrated that GSDMD‐mediated pyroptosis contributes directly to cisplatin‐induced hearing loss, whereas pharmacological inhibition or genetic deletion of GSDMD attenuates cochlear damage and auditory dysfunction [31]. The reciprocal changes in cleaved caspase‐1 and GSDMD‐N following G3BP1 manipulation, together with reduced IL‐18 release after G3BP1 overexpression, further support an association between G3BP1 and pyroptosis‐related inflammatory signalling.

Several mechanisms may potentially link G3BP1/SG responses to inflammasome signalling. SG formation has been reported to regulate factors such as DDX3X, potentially limiting NLRP3 inflammasome assembly and downstream ASC speck formation, caspase‐1 activation and cytokine maturation [32]. SG‐associated stress responses may also influence mitochondrial ROS, mtDNA release, or K^+^ efflux and thereby modulate inflammasome activation [33, 34]. These mechanisms remain hypothetical in ARHL and will require direct assessment of SG formation, DDX3X localization, ASC speck formation and mitochondrial function.

Several limitations should be acknowledged. Young animals were not included in the original RNA‐sequencing experiment, and a complete age × strain transcriptomic design is therefore still required. In addition, the functional effects of G3BP1 were primarily examined in HEI‐OC1 cells, and in vivo gain‐ or loss‐of‐function studies will be required to determine whether G3BP1 directly modifies auditory decline during aging.

Overall, our findings identify G3BP1 as a stress‐responsive regulator associated with strain‐dependent susceptibility to ARHL and suggest that increased G3BP1 may restrain caspase‐1/GSDMD‐associated inflammatory signalling. Recent advances in inner‐ear gene therapy have demonstrated that disease‐relevant molecular pathways within the cochlea can be therapeutically manipulated. For example, AAV‐mediated base editing has achieved substantial and sustained auditory recovery in a DFNA15 mouse model [35]. Although hereditary hearing loss and ARHL differ fundamentally in aetiology, these findings highlight the therapeutic accessibility of cochlear molecular pathways and support further investigation of G3BP1 and SG‐associated stress responses as potential targets for enhancing cochlear resilience during aging.

## 5 Conclusion

This study identifies G3BP1 as a stress‐responsive regulator associated with strain‐dependent susceptibility to ARHL. Cochlear transcriptomic analysis revealed enrichment of stress granule‐ and translation stress‐related signatures in the more susceptible strain, while cellular experiments showed that prolonged oxidative stress was accompanied by increased G3BP1 expression and enhanced caspase‐1/GSDMD‐associated activation. Functionally, G3BP1 knockdown aggravated, whereas G3BP1 overexpression attenuated pyroptosis‐associated activation and reduced IL‐18 release. Together, these findings support a protective role of G3BP1 in cochlear stress adaptation. Further studies are needed to determine whether this effect is specifically mediated through stress granules and to validate its role in vivo during ARHL progression.

## Author Contributions

**Chen Li:** writing – original draft, conceptualization, methodology, project administration, investigation. **Yuan Cao:** methodology. **Jun Zhou:** data curation. **Yurun Chen:** supervision. **Qi Tang:** validation. **Shuyi Wang:** software. **Zhiyuan Wu:** formal analysis. **Shu Wang:** visualization. **Zixin Zhang:** methodology. **Mengyao Xie:** supervision. **Hong Huo:** supervision, funding acquisition, resources. **Hua Yang:** writing – review and editing, funding acquisition, resources.

## Funding

The author(s) disclosed receipt of the following financial support for the research, authorship and/or publication of this article: this project was supported by the CAMS Innovation Fund for Medical Sciences (CIFMS) (2025‐I2M‐C&T‐B‐007), the Beijing Natural Science Foundation (7262095), the Non‐profit Central Research Institute Fund of Chinese Academy of Medical Sciences (2024‐RW320‐06) and the Fundamental Research Funds for the Central Universities, Peking Union Medical College (3332024128).

## Disclosure

During the preparation of this work the author(s) used ChatGPT in order to improve language. After using this tool/service, the author(s) reviewed and edited the content as needed and take(s) full responsibility for the content of the published article.

## Conflicts of Interest

The authors declare no conflicts of interest.

## Supporting information


**Supplementary Table 1.** Primer sequences used for quantitative real‐time PCR (qRT‐PCR).

## Data Availability

The data that support the findings of this study are available on request from the corresponding author. The data are not publicly available due to privacy or ethical restrictions.
