# Supplementary material for: Transcriptomics Implicates the G3BP1–SG Axis in Cochlear Hair‐Cell Pyroptosis During Age‐Related Hearing Loss
Source: J Cell Mol Med. 2026 Oct 11;30(19):e71403. doi: 10.1111/jcmm.71403 (PMC13654818; doi:10.1111/jcmm.71403)
Supplement: Supplementary file 1 [file JCMM-30-e71403-s001.docx]

**Supplementary table 1. Primer sequences used for quantitative real-time PCR (qRT-PCR).**

| Gene | Primer | Sequence (5′–3′) | Length (bp) |
| --- | --- | --- | --- |
| G3BP1 | F | GCTCAGCCGCGCAGATTTTA | 20 |
| G3BP1 | R | CTTGGTGTGGCAGTTGGTGA | 20 |
| Gapdh(M) | F | CATCACTGCCACCCAGAAGACTG | 23 |
| Gapdh(M) | R | ATGCCAGTGAGCTTCCCGTTCAG | 23 |

F, forward primer; R, reverse primer.
